# The Phylum Bryozoa as a Promising Source of Anticancer Drugs

**DOI:** 10.3390/md17080477

**Published:** 2019-08-17

**Authors:** Blanca Figuerola, Conxita Avila

**Affiliations:** 1 Institute of Marine Sciences (ICM-CSIC), Pg. Marítim de la Barceloneta 37-49, 08003 Barcelona, Catalonia, Spain; 2 Department of Evolutionary Biology, Ecology, and Environmental Sciences, and Biodiversity Research Institute (IrBIO), Faculty of Biology, University of Barcelona, Av. Diagonal 643, 08028 Barcelona, Catalonia, Spain

**Keywords:** antitumor compounds, marine natural products (MNPs), bioactivity, cytotoxicity, marine invertebrates

## Abstract

Recent advances in sampling and novel techniques in drug synthesis and isolation have promoted the discovery of anticancer agents from marine organisms to combat this major threat to public health worldwide. Bryozoans, which are filter-feeding, aquatic invertebrates often characterized by a calcified skeleton, are an excellent source of pharmacologically interesting compounds including well-known chemical classes such as alkaloids and polyketides. This review covers the literature for secondary metabolites isolated from marine cheilostome and ctenostome bryozoans that have shown potential as cancer drugs. Moreover, we highlight examples such as bryostatins, the most known class of marine-derived compounds from this animal phylum, which are advancing through anticancer clinical trials due to their low toxicity and antineoplastic activity. The bryozoan antitumor compounds discovered until now show a wide range of chemical diversity and biological activities. Therefore, more research focusing on the isolation of secondary metabolites with potential anticancer properties from bryozoans and other overlooked taxa covering wider geographic areas is needed for an efficient bioprospecting of natural products.

## 1. Introduction

Most bioactive secondary metabolites have been isolated from species inhabiting terrestrial environments, although oceans cover >70% of the Earth’s surface and marine natural products (MNPs) generally show higher incidence of significant cytotoxic activity [1,2]. It is well known that most sessile marine invertebrates produce active natural products for a variety of ecological roles, such as defense against predators, parasites and infections, and/or competition, it being thus that aquatic environments are important potential sources of compounds [3]. A large number of these chemicals have pharmacological activity for their interaction with receptors and enzymes, and thus are continuously gaining interest in the biomedical field [4]. In particular, there is an increasing demand on the development of new anticancer drugs as cancer is one of the deadliest diseases worldwide.

In recent decades, advances in scuba diving, deep-sea sample collection, and novel techniques in drug synthesis and aquaculture have promoted the discovery of an important number of compounds derived from marine organisms with potential anticancer properties [5,6]. One good example that the new technologies provide unprecedented access to a previously untapped source of chemical diversity is the recent isolation of a variety of compounds from deep sea taxa which show cytotoxic properties toward a range of human cancer cell lines, even though most marine compounds have still been isolated from shallow fauna [7]. Eight anti-cancer drugs based on MNPs have already been approved for human use, although only a small proportion (one out of 5000–10,000) of the new synthetic molecules becomes a commercial drug due to toxicity [8]. Among these anti-cancer drugs, Eribulin is an analogue of the MNP halichondrin-B, which induces apoptosis of cancer cells and was isolated from the sponge genus *Halichondria* Fleming, 1828 and used for the treatment of liposarcoma and breast cancer [9,10]. Ziconotide is a toxin derived from the mollusk *Conus magus* Linnaeus, 1758 which acts as a painkiller by blocking calcium channels in pain-transmitting nerve cells [11]. Brentuximab vedotin is an antibody drug conjugate for which the payload was isolated from mollusk *Dolabella auricularia* (Lightfoot, 1786) and is an antibody-drug conjugate used to treat Hodgkin’s lymphoma and systemic anaplastic large cell lymphoma (ALCL) [12,13]. Cytarabine is used to treat acute myeloid leukemia, acute lymphocytic leukemia, chronic myelogenous leukemia, and non-Hodgkin’s lymphoma isolated from the sponge *Tectitethya crypta* (de Laubenfels, 1949) [14]. Trabectedin is a drug isolated from the tunicate *Ecteinascidia turbinata* Herdman, 1880 which is used for the treatment of advanced soft tissue sarcoma [15,16]. The vast majority of studies on assessment of anticancer properties of marine-invertebrate-derived compounds have focused on different invertebrate groups, such as sponges and corals [17,18]. By contrast, few MNPs, and in particular good candidates for anticancer drugs, have been isolated from bryozoans despite many of them having been shown to be bioactive and/or to have unique chemical structures [19,20]. 

Bryozoa (sea mats, moss animals, or lace corals), a phylum of aquatic, filter-feeding invertebrates, are abundant, speciose, ubiquitous, and important members of many benthic communities from the intertidal to the deep sea in a variety of marine habitats [21]. Over 6000 extant species are known, with new taxa being continuously described, particularly in regions which have been previously inaccessible (e.g., deep sea and Antarctica) [22,23,24,25,26]. Species are almost exclusively colonial and their colonies are generally sessile, developing a broad spectrum of forms (ranging from encrusting sheets to erect branching chains), which provide habitats for a wide range of small invertebrates and microorganisms [21]. The individual functional units (modules) of colonies are called zooids. This phylum is traditionally organized into three classes: Phylactolaemata (freshwater), Gymnolaemata (mostly marine), and Stenolaemata (marine). The Gymnolaemata contains two orders: Cheilostomatida and Ctenostomatida. Recent molecular sequence data has shown that Phylactolaemata is the sister group to Gymnolaemata and Stenolaemata ([27]). The Gymnolaemata and Stenolaemata comprise bryozoans with a calcified skeleton, except for ctenostomes. 

Bryozoans are excellent sources of pharmacologically interesting substances, including alkaloids and polyketides with diverse biological activities (e.g., antimicrobial and antipredation [20,28]). Regarding unexplored regions, our recent studies on chemo-ecological interactions of a range of bryozoan species from different Antarctic locations have reported a variety of ecological roles of their lipophilic and hydrophilic extracts. These activities include defensive strategies against microorganisms [29,30] and against abundant and ubiquitous sympatric predators [30,31,32], as well as cytotoxicity against a common sea urchin [33,34], reducing its reproductive success. Therefore, more effort is required to isolate and characterize the secondary metabolites involved in these chemical interactions for their potential in pharmacological applications. This phylum has received little attention until now, with most studied species possessing erect, foliose, and large colonies and belonging to the order Cheilostomatida. Some of the reasons for these scarce studies may include the usually insufficient biomass of bryozoan samples to allow for the isolation of secondary metabolites, which is related to the fact that many species are heavily calcified, and also the technical difficulties for collecting the specimens due to their often encrusting growth and difficult taxonomy (e.g., lack of taxonomic expertise and laborious and time-consuming identification under the microscope) [35]. More efforts should be thus devoted to studying its taxonomy and to collect uncalcified bryozoans (ctenostomes) and encrusting species, which regularly have to compete for available surfaces, and thus could be expected to be a rich source of natural products [20]. 

The origin of the bioactive compounds in marine invertebrates is mostly unknown, although it has often been demonstrated to originate either from de novo biosynthesis, from the diet, or from symbiotic microorganisms [36]. In bryozoans, the origin of bryostatins has been traced to bacterial symbiont *Endobugula sertula* [37,38], but it is still unknown for the rest of the compounds.

The purpose of this review is to showcase the secondary metabolites with potential anticancer properties isolated from 14 marine cheilostome and two ctenostome bryozoans. In particular, we describe the cytotoxic activity against cancer cells of different class of compounds including alkaloids, sterols, ceramides, and polyketides, namely the bryostatins, which are the most well-known and promising secondary metabolites in cancer chemotherapy produced by marine organisms. We generally report their biological activity using IC_50_ values (the concentration of a drug that is required for 50% inhibition), considering active the compounds with IC_50_ values smaller than 10 μM [39], although some original papers cited here report higher values as active following different criteria. It must be emphasized that the cytotoxic compounds described here can therefore also possess cytotoxicity against normal cells. The bioselective compounds, which show higher ranges of growth inhibitory effects in cancer in comparison to normal cells, are good candidates to become potential anticancer agents [40].

## 2. Cytotoxic Compounds from Marine Bryozoans with Activity against Cancer Cell Lines

The MNPs from bryozoans are diverse and display a wide variety of activities, from which here we review the cytotoxic activity against cancer cells.

### 2.1. Alkaloids

**Figure 1:**
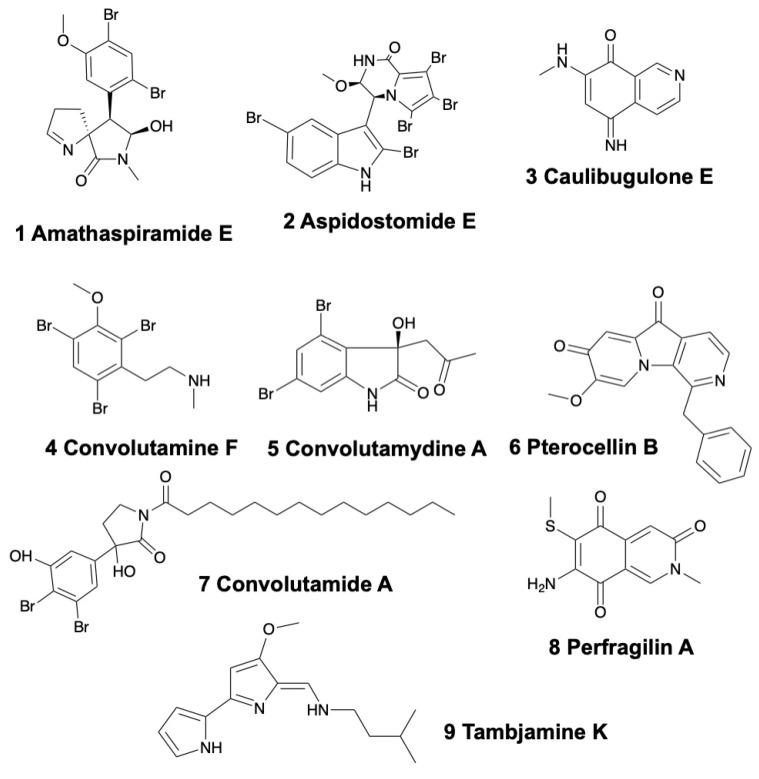
Structures of selected alkaloids from bryozoans.

Alkaloids are the most common class of natural products isolated from bryozoans with a unique structural and bioactive diversity (Figure 1). Therefore, these secondary metabolites have a huge potential as new drugs [19].

#### 2.1.1. Amathaspiramides

Amathaspiramides A–F, a series of six dibrominated alkaloids, were isolated from *Amathia wilsoni* Kirkpatrick, 1888 (Vesiculariidae) by Morris and Prinsep [41]. This specimen was collected from Barrett Reef in Wellington Harbor (off the North Island of New Zealand). Amathaspiramides were assayed for P-388 murine lymphocytic leukemia but none of the compounds were active (IC_50_ value > 12.5 µg/mL). These compounds, together with four analogues, were tested in vitro for antiproliferative activity against four human cancer cell lines (HCT-116 (colon cancer), PC-3 (prostate cancer), MV4-11 (acute myeloid leukemia), and MiaPaCa-2 (pancreas cancer)). Amathaspiramide C only exhibited antiproliferative activity (<10 µM) against one (MiaPaCa-2) of the four cancer cell lines (IC_50_ values of 63, 80, 64, and 5.8 µM, respectively), while Amathaspiramide A (HCT-116, PC-3, MV4-11, and MiaPaCa-2: IC_50_ values of 46, 67, 48, and 14 µM, respectively) and Amathaspiramide E (**1**) (IC_50_ values of 29, 81, 55, and 15 µM, respectively) did not show activity in any of all four cell lines [42]. This study demonstrated the importance of the amine or imine substructure on the pyrrolidine moiety and the 8*R* stereochemistry on the *N*-acyl hemiaminal moiety for the antiproliferative activity of amathaspiramides.

#### 2.1.2. Aspidostomides

Aspidostomides A–H are a series of bromopyrrole alkaloids derived from either bromotryptophan or bromotyrosine and isolated from the Patagonian bryozoan *Aspidostoma giganteum* (Busk, 1854) (Aspidostomatidae) by Patiño et al. [43]. Remarkably, there have been no previous reports of secondary metabolites from this family and this study is the first report on the chemistry of a bryozoan species from the Patagonian region [43]. Two cheilostome specimens were collected by trawling (60–100 m) in the Gulf of San Jorge (Argentina) having a wide distribution along South America and Antarctic regions. Aspidostomide E (**2**) exhibited moderate inhibitory activity towards the 768-O renal carcinoma cell line (IC_50_ value 7.8 μM).

#### 2.1.3. Brominated Alkaloids

A bromated alkaloid (7-bromo-2,4(1H,3H)-quinazolinedione) from the cheilostome *Cryptosula pallasiana* (Moll, 1803) (Cryptosulidae) collected off Huang Island (Qingdao, China) by Tian and co-workers did not show strong cytotoxicity against human myeloid leukemia HL-60 cells (IC_50_ value 11.87 μg/mL) [44]. 

#### 2.1.4. β-Carboline Alkaloids

The crude extracts of the cheilostomes *Paracribricellina* (*Catenicella*) *cribraria* (Busk, 1852), collected at a 14 m depth on Cape Vlamingh (Rottnest Island, Western Australia), and *Paracribricellina* (*Cribricellina*) *cribraria* (Busk, 1852) (Catenicellidae), collected at a 15 m depth at Poor Knights Islands (The Tunnel, North Wall, New Zealand), exhibited relatively potent cytotoxicity against an NCI-60 cell tumor assay (the most sensitive cell line subpanel was the melanoma, where the median lethal concentration (LC_50_) values of eight out of nine cell lines were similar to, or slightly less, than the mean panel LC_50_ of 19 μM). The compound 1-vinyl-8-hydroxy-β-carboline was responsible for the activity against the NCI-60 cell tumor in both species [45]. This compound, the major cytotoxic component from the latter species, was previously isolated by Prinsep and co-workers [46] by scuba diving off Sugar Loaf, Kaikoura (off the South Island of New Zealand), showing cytotoxicity against P-388 (IC_50_ value of 0.1 μg/mL). Other β-carboline alkaloids were also isolated from the same species, showing different degrees of biological activity in the P-388 cytotoxicity assay: the IC_50_ value of 1-vinyl-8-methoxy-β-carboline and pavettine were determined to be 0.1 μg/mL, while that of compound 1-vinyl-8-acetoxy-β-carboline was 0.67 μg/mL [46]. The IC_50_ values of 1-ethyl-4-methylsulfone-β-carboline, 1-ethyl-8-hydroxy-β-carboline, and l-ethyl-8-methoxy-β-carboline were both greater than 12.5 μg/mL. A previously described compound, 6-hydroxyharman, and a new β-carboline alkaloid, 8-hydroxyharman, from *P. cribraria*, collected from Lighthouse Reef Point (Moeraki, east coast of the South Island, New Zealand), exhibited relatively weak cytotoxicity against P-388 cells with an IC_50_ more than 12.5 μg/mL [47]. Moreover, another β-carboline alkaloid, 5-bromo-8-methoxy-1-methyl-β-carboline, was isolated for the first time from the cheilostome *Pterocella vesiculosa* (Lamarck, 1816) (Catenicellidae) collected from the Alderman Islands (off the North Island, New Zealand) by Till and co-workers [48]. The alkaloid displayed relatively moderate cytotoxicity against P-388 cells with an IC_50_ of 5.089 μg/mL and also displayed inhibitory action against the Gram-positive bacterium *Bacillus subtilis* and the fungi *Candida albicans* and *Trichophyton mentagrophytes* with minimal infecting dose (MID) of 2–4, 4–5, and 4–5 μg/mL [48]. It has been demonstrated that the vinyl substituent at C-1 or bromine at C-5 is important for the cytotoxicity against P-388 [48].

#### 2.1.5. Caulamidines

Caulamidine A and B, heterocyclic alkaloids with a 2,6-naphthyridine core and fused by dihydroindole-derived and tetrahydroquinoline-derived systems, were isolated from the cheilostome *Caulibugula intermis* Harmer, 1926 (Bugulidae). Caulamidine A did not display cytotoxicity in an NCI-60 cell screen with a single dose (40 μM). Both compounds exhibited antimalarial activity towards *Plasmodium falciparum* with IC_50_ values from 8.3–12.9 μM [49].

#### 2.1.6. Caulibugulones

Caulibugulones A–F are alkaloids isolated from specimens of the *C. intermis* collected at a depth of 33 m in the south Pacific off Palau by Milanowski and co-workers [50]. Caulibugulones A–D possess an isoquinoline-5,8-dione carrying a substituted amino group at position C-7 and substitution at C-6 by hydrogen, bromine, or chlorine. Caulibugulones E (**3**) and F are analogues of caulibugulone A carrying an imine group at position C-5 and were the first compounds with an isoquinoline iminoquinone skeleton to be isolated from a natural source [50]. All these alkaloids showed cytotoxicity against the murine IC-2^wt^ tumor cell line in vitro with IC_50_ from 0.03 to 1.67 μg/mL, although caulibugulone E was the most potent. A series of isoquinoline quinones were isolated from marine sponges. Similar compounds were isolated from bacterial sources, suggesting a bacterial origin. These compounds displayed antitumor activities as well ([50] and references therein).

#### 2.1.7. Convolutamides, Convolutamydines, and Convolutamines

Convolutamides A–F are alkaloids, possessing an *N*-acyl-γ-lactam moiety with a dibromophenol group, isolated from the ctenostome *Amathia convoluta* (Lamarck, 1816) (Vesiculariidae) by Zhang and co-workers [51]. The specimens were collected off the Northeastern Gulf of Mexico in Florida (US). The mixture of convolutamides A (**7**) and B displayed cytotoxicity against L-1210 murine leukemia cells and human epidermoid carcinoma (KB) cells (IC_50_ values of 4.8 and 2.8 μg/mL, respectively) [51]. 

Convolutamydines A–D belong to a class of alkaloids isolated from *A. convoluta* collected in the same region by Kamano and co-workers [52]. Convolutamydine A (**5**) was the first example of a compound with oxindole for marine bryozoans. This compound (1,4,6-dibromo-3-hydroxy-3-(2-oxopropyl)-2-indolinone) showed potent activity in the differentiation of HL-60 human plomyelocytic leukemia cells at concentrations of 0.1–25 μg/mL [52]. Further investigation by Zhang et al. [53] led to the isolation of three new dibromohydroxyoxindole derivatives, convolutamydines B–D. Given the small amounts of convolutamydines C and D, their biological evaluation could not be achieved [53]. 

Convolutamines A–G (brominated β-phenylethylamine alkaloids) and lutamides A and C (2,4,6-tribromo-3-methoxyphenethylamine alkaloids) were also isolated from the Floridian *A. convoluta* [54]. Convolutamines A, C, and F exhibited inhibition against adriamycin (ADM)-resistant P-388/ADM (IC_50_ values 7.0, 3.0, and 9.5 μg/mL, respectively) and vincristine (VCR)-resistant P-388/VCR (IC_50_ values 3.0, 1.4, and 8.0 μg/mL, respectively) [54]. Convolutamines B and D also exhibited cell growth inhibitory activity against P-388 with IC_50_ values of 4.8 and 8.6 μg/mL, respectively [55] and convolutamine F (**4**) against its vincristine-resistant KB/VJ-300 cells with an IC_50_ value of 9.6 μg/mL [56]. Convolutamine F also displayed inhibitory effect for cell division of fertilized sea urchin eggs with an IC_50_ value of 82 μg/mL [56]. Convolutamines I–J, isolated from the Southern Ocean bryozoan *A. tortuosa* Tenison-Woods, 1880, were recently validated as potential ATP competitive inhibitors [57]. Lutamides A and C exhibited inhibition against KB/VJ300 cells (IC_50_ values 7.5 and 6.5 μg/mL, respectively) and lutamide C against P-388/VCR (IC_50_ value 4.8 μg/mL) and in the presence of ADM or VCR whose concentration did not affect growth of the cells examined [54].

#### 2.1.8. Eusynstyelamides

Eusynstyelamides are alkaloids isolated from different marine organisms such as bryozoans and ascidians [58]. The brominated tryptophan-derived ent-eusynstyelamide B and three new derivatives, eusynstyelamides D, E, and F, were isolated from the Arctic cheilostome *Tegella cf. spitzbergensis* (Bidenkap, 1897) (Calloporidae) by Tadesse and co-workers [58], being the first report of bioactive metabolites from this genus. The bryozoan specimen was collected off the Bear Islands (North Atlantic) at 59 m depth. Two compounds, eusynstyelamide D and E, did not exhibit activity against the human melanoma A-2058 cell line (IC_50_ values 57 and 114.3 μg/mL, respectively) [58]. Eusynstyelamide B, together with its two isomers eusynstyelamide A and C, were previously isolated from the Australian ascidian *Eusynstyela latericius* (Sluiter, 1904) collected using scuba from the waters around Hixson Island and Rib Reef. These compounds were found to be nontoxic toward the three human tumor cell lines MCF-7 (breast), SF-268 (central nervous system), and H460 (lung) at concentrations of up to 32 mM despite exhibiting inhibitory activity against neuronal nitric oxide synthase and modest antibacterial activity [59]. Eusynstyelamide D, almost identical to eusynstyelamide A, was isolated from another ascidian species *E. misakiensis* (Watanabe and Tokioka, 1972). The compound was nontoxic towards human colon cancer cell line HCT-116 [60]. 

#### 2.1.9. Perfragilins

Perfragilins A (**8**) and B are isoquinoline quinones isolated from the cheilostome *Biflustra perfragilis* MacGillivray, 1881 (Membraniporidae) and collected using scuba at Rapid Bay (South Australia). Both perfragilins displayed cytotoxic activity to murine leukemia cell (P-388) lines (median effective dose (ED_50_) values of 0.8 and 0.07 μg/mL, respectively) [61]. Their structural relationship to the alkaloid mimosamycin, isolated from the terrestrial actinomycete *Streptomyces lavendulae* [62] and the sponge *Haliclona* (*Reniera*) sp., collected in a marine lake in Palau, Western Caroline Islands [63], as well as *Niphates* (*Xestospongia*) *caycedoi* (Zea and Van Soest, 1986) collected from Sand Island (Suva Harbor, Fiji), at a 2 m depth, suggests that perfragilins are also of bacterial origin [61]. 

#### 2.1.10. Polycyclic Indole Alkaloids

The primary source of these alkaloids are four bryozoan species belonging to the family Flustridae: *Chartella papyracea* (Ellis and Solander, 1786), *Securiflustra securifrons* (Pallas, 1766), *Hincksinoflustra denticulata* (Busk, 1852) and *Flustra foliacea* (Linnaeus, 1758). However, only some alkaloids have been shown to be active against tumor cell lines until now. Chartellines A–C and chartellamides A–B were isolated from *C. papyracea* in the Roscoff region of France by Anthoni and co-workers [64,65], being the first examples of polycyclic indole alkaloids in marine bryozoans. Chartelline A, the first compound isolated from this species with a penta-halogenated indole containing a β-lactam ring, was inactive against leukemia cells in the NCI test [65]. Three new halogenated, hexacyclic indole-imidazole alkaloids, securamines H–J, together with the previously reported compounds securamines C and E, were isolated from the Arctic bryozoan *S. securifrons* collected off the coast of Hjelmsøya (Norway) by Hansen and co-workers [66]. Securamines C, E, and H–J were evaluated for their cytotoxic activity against human cancer cell lines A-2058 (skin), HT-29 (colon), and MCF-7 (breast), as well as against non-malignant human MRC-5 lung fibroblasts. Securamines C, E, H, and I were found to affect cell viability, with H, I, and E being the most potent and with IC_50_ values ranging from 1.4 to 10 μM [66]. While a crude extract of *F. foliacea* collected in the southeastern North Sea at a water depth of 33–45 m by Lysek et al. [67] was not cytotoxic, their purified compounds displayed activity against human colon cancer cell line HCT-116. In particular, a new deformylflustrabromine showed the strongest cytotoxicity (IC_50_ value 5.8 μM) and flustramines A and D and dihydroflustramine C did not display cytotoxicity (IC_50_ value 26 μM) [67]. 

#### 2.1.11. Pterocellins

Pterocellins A–F are a series of alkaloids based on a 4-pyridone group and a pyridine group bound together through a five membered ring which appears to be unique to the cheilostome *Pterocella vesiculosa* [68,69]. Specifically, pterocellins A and B (**6**) were isolated from an organic extract of this bryozoan species from the Hen and Chicken Islands (off the North Island of New Zealand) by Yao and co-workers [68]. Both compounds displayed relatively potent antitumor activity against the murine leukemia cell line P-388 in vitro with IC_50_ values of 0.477 and 0.323 μg/mL, respectively, apart from antibacterial and antifungal activities [68]. Moreover, the National Cancer Institute tested pterocellins A and B against a variety of human tumor cell types (leukemia, non-small cell lung, colon, central nervous system, melanoma, ovarian, renal, prostate, and breast cancers), exhibiting potent cytotoxicity overall (panel average values of GI_50_ (growth inhibition of 50%) 1.4 μM, TGI (tumor growth inhibition) 4.8 μM, and LC_50_ 17.0 μM for pterocellin A and GI_50_ 0.7 μM, TGI 2.1 μM, and LC_50_ 6.9 μM for pterocellin B) [68]. The most sensitive cell lines to pterocellins A and B were leukemia (CCRF-CEM: GI_50_ 0.05 μM, TGI 0.8 μM) and melanoma (MALME-3M: GI_50_ 0.03 μM, TGI 0.1 μM), respectively. Non-small cell lung (NCI-H23: GI_50_ 0.3 and 0.1 μM, TGI 1.0 and 0.3 μM, and LC_50_ 6.1 and 0.7 μM, respectively), melanoma (MALME-3M: GI_50_ 0.1 and 0.03, TGI 0.3 and 0.1 μM, and LC_50_ 0.8 and 0.3 μM, respectively; M-14: GI_50_ 0.2 and 0.1 μM, TGI 0.8 and 0.2 μM, and LC_50_ 4.6 and 0.5 μM, respectively; SK-MEL-5: GI_50_ 0.2 and 0.1 μM, TGI 0.3 μM, and LC_50_ 0.6 and 0.5 μM, respectively) and breast (MDA-MB-435: GI_50_ 0.2 μM, TGI 0.3 μM, and LC_50_ 0.6 μM, respectively, and MDA-N: GI_50_ 0.2 μM, TGI 0.4 and 0.3 μM, and LC_50_ 0.6 μM, respectively) were especially sensitive to both compounds. Only pterocellin A was tested in preliminary in vivo antitumor evaluation in a mouse hollow fiber assay given that the other derivate had a similar cytotoxicity profile. The results showed it was not effective, and it was discarded for the next stage of testing [35]. Also, this alkaloid was cytotoxic to Hela human cervical cancer cells, with an IC_50_ of 0.886 μg/mL [70]. Four new pterocellins (C–F) were isolated from another specimen posteriorly collected from the Alderman Islands (off the North Island of New Zealand). Pterocellins C–F displayed variable levels of activity against the Gram-positive bacterium *Bacillus subtilis* but only pterocellin D exhibited moderate activity against the P-388 cell line with an IC_50_ value of 4.773 μg/mL and against the dermatophyte *Trichophyton mentagrophytes* [69].

#### 2.1.12. Tambjamines

The tambjamines A–J are a 2-2′-bipyrrolic class of cytotoxic alkaloids isolated from bacteria and several marine invertebrate groups such as bryozoans, nudibranch mollusks and ascidians with a wide range of bioactive activities (e.g., antitumor and immunosuppressive activities) [71,72,73,74,75]. These compounds belong to the group of 4-methoxypyrrolic natural products and their structure is characterized by a pyrrole ring displaying a second pyrrole system at C-2, an enamine moiety at C-5, and a methoxy group at C-4. A new tambjamine K (**9**) together with the known tambjamines A and B were isolated from the cheilostome *Virididentula* (*Bugula*) *dentata* (Lamouroux, 1816) (Bugulidae) by Carbone and co-workers [76]. The bryozoan specimens were collected from the port of Horta at Faial island (Azores, Atlantic). Tambjamine K, the isopentenyl derivative of the co-occurring tambjamine A, displayed antiproliferative and cytotoxicity against tumor and non-tumor cell lines with an IC_50_ value against the human epithelial colorectal adenocarcinoma CaCo-2 cells within the nano-molar range (CaCo-2 cells: IC_50_ 3.5 × 10^−3^ μM; H9c2 cells: IC_50_ 2.7 μM). Due to the antitumor properties of this compound, Aldrich and co-workers [77] synthesized tambjamine K and a library of unnatural analogues which were more potent in viability, proliferation, and invasion assays than the natural product in multiple cancer cell lines, with minimal to no cytotoxicity on non-transformed cell lines. Also, tambjamine K was tested against human colon cancer HCT-116 and breast carcinoma MB-231 cell lines by the same team, being not active (IC_50_ 13.7 μM and IC_50_ 15.3 μM, respectively) [77].

#### 2.1.13. Terminoflustrindoles

Terminoflustrindoles A–C are a group of brominated akaloids isolated from the cheilostome *Terminoflustra* (*Chartella*) *membranaceotruncata* (Smitt, 1868) (Flustridae) and collected by Maltseva and co-workers [78,79] in the vicinity of the Marine Biological Station (Saint-Petersburg State University) Chupa Inlet, Kandalaksha Bay, the White Sea. Terminoflustrindole A was previously found to exhibit fungicidal activity [78] and was recently found to display antibacterial and cytotoxic activity against tumor (normal mice fibroblasts 3T3, transformed mice fibroblasts 3T3-SV40, human neuroblastome SK-N-SH, rat histioblastome C6, mice melanoma B-16, and histiotypic leukemia U-937 cells) and normal cells at concentrations of 10 μM, although the effect on normal cells was significantly less [79].

### 2.2. Lactones

**Figure 2:**
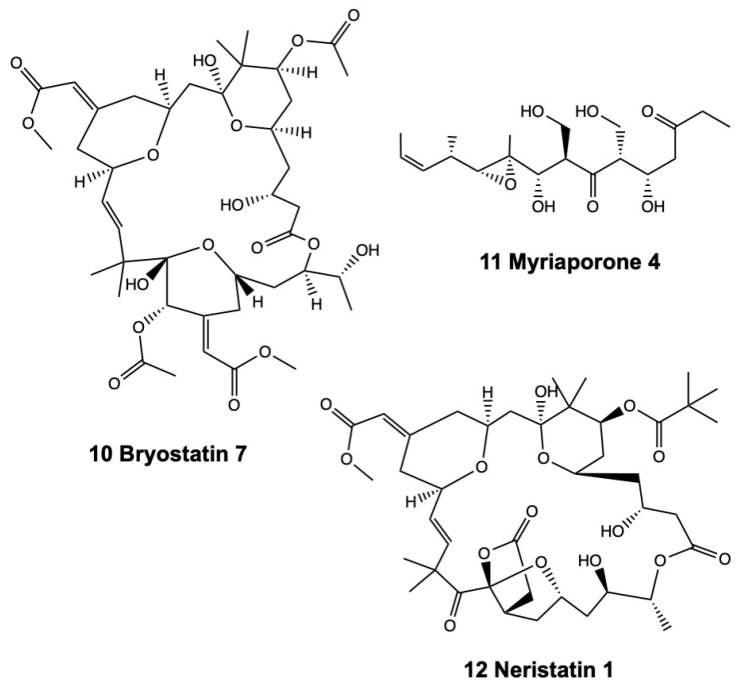
Structures of selected lactones and a ceramide from bryozoans.

Macrocyclic lactones are a relevant class of secondary metabolites with antitumor activity (Figure 2). Bryostatin-1 was the first macrocyclic lactone identified from *Bugula neritina* (Linnaeus, 1758) (Bugulidae) (72). 

#### 2.2.1. Bryostatins

Bryostatins are the most known class of marine-derived compounds from bryozoans and the most promising compound candidates as anticancer agents due to their low toxicity and antineoplastic activity. Since the discovery of bryostatin-1 by Pettit and collaborators in 1982 [80], bryostatins 2–18 have been described by Pettit et al. [81,82,83,84,85,86,87,88,89,90] and a wide range of studies have been published focusing on their potential use in cancer chemotherapy. This class of highly oxygenated macrolides are complex polyketides based on the bryopyran ring system and the main developments in their structure-activity relationship and biology have been recently summarized by several reviews [91,92,93]. It has been demonstrated that the structures with α methyl at C-28 and α hydroxyl at C-9 play a significant role in their potent cytotoxicities [19]. Bryostatins 1-18 have been identified and tested for antitumor activity [91]. Among in vitro and in vivo anticancer effects against a range of tumor lines, bryostatins have been shown to display activity against histiocytic lymphoma cell U-937, human leukemia HL-60, lymphocytic leukemia P-388, melanoma B-16, murine melanoma K1735-M2, prostate cancer cells LNcaP and M-5076 reticulum cell sarcoma [92,93]. Apart from their antitumor properties, these compounds can be used to treat other diseases, as bryostatins improve memory and learning (e.g., Alzheimer’s disease, depression, and traumatic brain injury) [94]. 

Bryostatins are able to selectively modulate the function of several protein kinase C (PKC) enzymes, which possess an important role in the regulation of cell growth and death. In particular, their anti-tumor effects are the consequence of binding to the PKC, whose activation by phorbol esters promotes the growth of tumor cells but whose interaction with bryostatins produces antineoplastic activity. In particular, bryostatin-7 (**10**), the first member of the bryostatins to be synthesized by Kageyama and co-workers [95], exhibits the most potent binding affinity to PKC and Tian et al. [19] has suggested that this compound could therefore be an effective substitute for bryostatin-1. Bryostatins 1, 5, and 8 promote the activation of PKC for a short period followed by its deregulation, leading to growth inhibition, cell differentiation, and programmed cell death [91,96]. It has been demonstrated that structures with a 20-membered macrolactone ring are essential for good PKC binding activity, a C-(3)-hydroxyl with (R)-stereochemistry and a free hydroxyl at C-26 are significant for a high enzyme affinity, and the structure of the A-ring at the C7-C9 region is needed for displaying antitumor activity [92]. 

Bryostatin-1, a highly oxygenated macrolide with a unique polyacetate backbone, is the most studied compound, and was first isolated and characterized by Pettit [80] from the cheilostome *B. neritina*. This temperate intertidal bryozoan species forms chitinous, branching colonies that are often found attached to vessel hulls and docks across the globe. This compound has been isolated from different colonies in a range of locations such as California and the Gulf of Mexico. Apart from being a PKC inhibitor, this bioactive compound has been shown to induce differentiation and promote apoptosis in various tumor cell lines [96], immunomodulatory properties (e.g., stimulation of cytokine production and activation of cytotoxic T lymphocytes) [97,98] and antitumor activity in preclinical models [99]. In addition, synergistic effects with a number of established oncolytic agents, including vincristine, paclitaxel, gemcitibine, and flavopyridol have been shown [92]. Therefore, bryostatin-1 is a promising compound against several tumor cell types, although it is still under investigation in Phase II clinical trials for cancer [93]. In addition, several analogues have been shown to have growth in vitro inhibitory activity against human cancer cell lines [5].

Bryostatin-5 showed a strong differentiation-inducing ability in human myeloid blast cells at concentrations of 10 nM [100] and inhibited the growth of murine melanoma K1735-M [101] and HL-60 leukemic cells at an optimal dose of 5–10 nM [102]. Bryostatin-19 isolated from *B. neritina* in the South China Sea displayed strong cytotoxic activity against the U-937 cell line with an in vitro ED_50_ value of 3.2 nmol/L [103]. 

These natural products with a broad range of biological activities have mainly been isolated from cheilostome and ctenostome bryozoans (e.g., *B. neritina* and *Amathia convoluta* (Lamarck, 1816) (Vesiculariidae), respectively), although other substances with different variations of the basic bryostatin structure have been reported in other marine organisms such as the sponge *Lissodendoryx isodictyalis* (Carter, 1882) and the ascidian *Aplidium californicum* (Ritter and Forsyth, 1917) [81,91,104]. Several studies have demonstrated that the bryostatins are more likely to be produced by the uncultured symbiotic bacterium *Endobugula sertula* [37,38] rather than being diet-derived compounds, or biosynthesized by the bryozoan itself. For example, some populations which do not harbor this symbiont do not have bryostatins either [105]. In laboratory experiments, *B. neritina* with the symbiont produced less bryostatins when the colonies were treated with antibiotics [37]. This bacterium is transmitted vertically to the larvae of *B. neritina* prior to their release from the adult [106]. It its documented that bryostatins cause the *B. neritina* larvae to be unpalatable to predators with a thousand-fold higher concentration found in the larvae than the adult colonies [38]. 

Syntheses of six bryostatins (1, 2, 3, 7, 9, and 16) have been published. A spectrum of bryostatin analogues have also been synthesized to replace bryostatins due to their scarcity from natural sources and the complexity of synthesis, modification, and extraction methods for their isolation. Importantly, these analogues retain biological activity despite their simplified structure [92]. Until now, several analogues have shown PKC inhibition activity with strong in vitro antitumor effects. For example, DeChristopher and co-workers demonstrated that the simplified bryostatin analogue 1, picolog, was a successful compound with stronger growth inhibition of MYC-induced lymphoma in vitro compared with bryostatin-1 at concentrations from 1 nM–10 μM ([107]; also see the review by Ruan et al. [92]). The same team showed that this analogue is effective in vivo in an animal cancer model.

#### 2.2.2. Neristatin 1

Neristatin 1 (**12**), a macrocyclic lactone, was isolated from the cheilostome *B. neritina*. Specimens were collected from the Gulf of Mexico coast of Florida (US). This compound is similar to the bryostatins [108], exhibiting weak activity (ED_50_ = 10 μg/mL) against the P-388 leukemia cell line [109].

#### 2.2.3. Myriaporones

Myriaporones are polyketide-derived metabolites isolated from the Mediterranean cheilostome *Myriapora truncata* (Pallas, 1766) (Myriozoidae). Isolation and structural determination of the Myriaporones 1–4 were achieved from a specimen collected from the Western Mediterranean Sea by Cheng and co-workers [110]. Given that myriaporones 3 (hemiketal, myriaporone 3) and 4 (hydroxy ketone) (**11**) are isomers in equilibrium, their structures were determined by analysis of a mixture which showed 88% inhibition against murine leukemia L-1210 cells at 0.2 μg/mL [110]. 

#### 2.2.4. Other Lactones

A lactone was isolated from *Cryptosula pallasiana* collected from Huang Island (Qingdao, China) [111]. The compound showed stronger cytotoxicity against HL-60, human hepatocellular carcinoma Hep-G2, and human gastric carcinoma SGC-7901, with IC_50_ values ranging from 4.12 to 7.32 μM, than sterols and ceramides. 

### 2.3. Ceramides

Two sulfates of ceramides were isolated from the cheilostome *Watersipora cucullata* (Busk, 1854) (Watersiporidae). The specimen was collected in Aichi Prefecture (Japan). Both compounds were inhibitors of the principal target of anticancer drugs DNA topoisomerase I enzyme, with IC_50_ values of 0.4 and 0.2 μM, respectively [112].

Five ceramides, neritinaceramides A–E (2S,3R,3′S,4E,8E,10E)-2-(hexadecanoylamino)-4,8,10-octadecatriene-l,3,3′-triol, (2S,3R,2′R,4E,8E,10E)-2-(hexadecanoylamino)-4,8,10-octadecatriene-l,3,2′-triol, (2S,3R,2′R,4E,8E,10E)-2-(octadecanoylamino)-4,8,10-octadecatriene-l,3,2′-triol, (2S,3R,3′S,4E,8E)-2-(hexadecanoylamino)-4,8-octadecadiene-l,3,3′-triol, and (2S,3R,3′S,4E)-2-(hexadecanoylamino)-4-octadecene-l,3,3′-triol were isolated from *B. neritina* by Tian et al. [113]. This specimen was collected in Daya Bay (Shenzhen, China). All the compounds were inactive against HepG2, human gastric carcinoma SGC-7901, and NCI-H460 cells (IC_50_ > 47.3 μM) [113].

Later, Tian and co-workers isolated two new ceramides, (2S,3R,4E,8E)-2-(tetradecanoylamino)-4,8-octadecadien-l,3-diol and (2S,3R,20R,4E,8E)-2-(tetradecanoylamino)-4,8-octadecadien-l,3,20-triol, together with two known ceramides from *C. pallasiana* [111]. The specimen was collected at Huang Island (China). These compounds did not display cytotoxicity against HL-60, Hep-G2, and SGC-7901 (IC_50_ values from 21.13 to 58.15 μM). 

### 2.4. Sterols

Sterols have been shown to exhibit many bioactivities (e.g., antibacterial, antitumor, and anti-inflammatory) in marine taxa such as corals, sponges, and echinoderms, although the position and stereochemistry of hydroxyl or methoxyl groups between C-23 and C-25 in the side chain are characteristic for marine bryozoans (see review [19]). Tian et al. [19] have suggested that more studies on testing their cytotoxicity should be carried out in view of their potential medical applications. 

Two oxygenated sterols, namely, 3β,24(S)-dihydroxycholesta-5,25-dien-7-one and 3β,25-dihydroxycholesta-5,23-dien-7-one, were isolated from cheilostome *B. neritina* (Bugulidae) inhabiting Daya Bay (Shenzhen, China) by Yang and co-workers [114]. These compounds did not show cytotoxicity towards three human cancer cell lines, namely, HepG2, HT-29, and NCI-H460 (IC_50_ values between 22.58 and 53.41 μg/mL) [114]. Other two new sterols ((22E)-cholest-4,22-diene-3β,6β-diol and (23S,24R)-dimethylcholest-7-ene-3β, 5α,6β-triol), a sterol reported for the first time from natural sources ((22E,24S)-24-methylcholest-4,22-diene-3β,6β-diol), a known steroid glycoside, and six known sterols were also isolated from B. neritina in the same region. All compounds were inactive against human tumor cell lines Hep-G2, NCI-H460, and SGC-7901 (IC_50_ > 36.6 μM) [115].

Thirteen sterols were isolated from the cheilostome *Cryptosula pallasiana* (Cryptosulidae) collected off Huang Island (Qingdao, China) by Tian et al. [116]. Three of these sterols were also isolated from the sponge *Cliona viridis* (*copiosa*) (Schmidt, 1862) (Clionaidae), collected in the Bay of Naples (Italy) at a depth of 15 m [117] and the deep-water sponge *Stelodoryx chlorophylla* Lévi, 1993 (Myxillidae), collected south of New Caledonia at a depth of 600–540 m [118]. Among the sterols, seven of them ((23E)-25-methoxycholesta-5,23-dien-3β-ol, (22E)-7β-methoxy-cholesta-5,22-dien-3β-ol, 7β-methoxy-cholest-5-en-3β-ol, (23E)-3β-hydroxy-27-norcholesta-5,23-dien-25-one, (23Z)-cholesta-5,23-diene-3β,25-diol, (22E)-3β-hydroxycholesta-5,22-dien-7-one, and (22E)-3β-hydroxy-24-norcholesta-5,22-dien-7-one) did not exhibit cytotoxic effects against human myeloid leukemia HL-60 cells (IC_50_ values from 14.73 to 22.11 μg/mL) [116]. 

A new sterol, (23R)-methoxycholest-5,24-dien-3β-ol, together with three known sterols, were isolated from *C. pallasiana* collected off Huang Island (China) [111]. All compounds were evaluated for their cytotoxicity against human tumor cell lines HL-60, Hep-G2, and SGC-7901, resulting in not being cytotoxic (IC_50_ values ranging from 12.34 μM to 18.37 μM). 

## 3. Other Compounds

A new antiangiogenic (inhibitor of the proliferation of endothelial cells) compound, bryoanthrathiophene, 5,7-dihydroxy-1-methyl-6-oxo-6*H*-anthra[1,9-*bc*]thiophene, together with two known compounds, 5,7-dihydroxy-1-methoxycarbonyl-6-oxo-6*H*-anthra[1,9-*bc*]thiophene and 1,8-dihydroxyanthraquinone, were isolated from bryozoan *Watersipora subtorquata* (d’Orbigny, 1852) (Watersiporidae) [119]. The specimen was collected in Tsutsumi Island (Fukuoka, Japan) at depths of 5–10 m. The three compounds were evaluated for antiangiogenic activity on basic fibroblast growth factor (bFGF)-induced proliferation of bovine aortic endothelial cells (BAEC). Bryoanthrathiophene was the most active compound with an IC_50_ of 0.005 μM, being thus potentially able to be used as treatments for cancer and angiogenesis-dependent diseases such as diabetic retinopathy and arthritis.

Three aromatic compounds, *p*-methylsulfonylmethyl-phenol, *p*-hydroxybenzaldehyde, and methylparaben, were isolated for the first time from the bryozoan species *C. pallasiana* apart from the alkaloids previously mentioned [44]. The new natural product *p*-methylsulfonylmethyl-phenol was evaluated for cytotoxicity against HL-60 cells and it appeared to be inactive. 

## 4. Active Extracts

The anti-cancer activity of an extract from the cheilostome *Schizoporella unicornis* (Johnston in Wood, 1844) (Schizoporellidae), inhabiting the Arabian Gulf and the Gulf of Oman, was tested in a Michigan Cancer Foundation (MCF-7) cell line breast adenocarcinoma model. The extract did not display anti-cancer activity (IC_50_ = 97 μg/mL). Further studies should be carried out to identify the compound/s involved [120].

An organic extract from the Arctic bryozoan *Alcyonidium gelatinosum* (Linnaeus, 1761), collected at Hopenbanken, off the coast of Edgeøya (Svalbard), was recently shown to inhibit the viability of the human melanoma cancer cell line A-2058, leading to the evaluation of the chemical constituents of the organic extract and the further isolation of ponasterones (ecdysteriods) for the first time in a bryozoan (ponasterone F and ponasterone A) by Hansen and co-workers [121]. As ecdysteroids are arthropod steroid hormones controlling molting (ecdysis), the authors hypothesized that these compounds could be used by the bryozoan to reduce fouling on their colonies. In fact, molting has been reported in the same genus (*A. sanguineum* Cook, 1985) and in the free-living bryozoan species *Cupuladria doma* (d’Orbigny, 1853) under conditions of heavy fouling colonies [122]. The compounds were posteriorly assayed for cytotoxic properties against A-2058 and the non-malignant human fibroblasts MRC-5, resulting in no affection of the survival of these cell lines at concentrations of up to 215 and 223 μM, respectively [121]. Further isolation of compounds is thus necessary to identify the compound(s) responsible for the bioactivity observed.

## 5. Future Research Directions

This review discusses a wide array of natural products of diverse nature isolated from a range of bryozoan species that have shown potential as cancer therapies: alkaloids, lactones, ceramides, sterols and other compounds, and active extracts (Table 1). Some of these MNPs may also have a variety of biotechnological properties (e.g., antibacterial and antifungal activities). Bryozoa is thus a promising source for cytotoxic/anticancer agents, as well as antibiotics. Their active compounds have been covered in a series of reviews by Blunt et al. (e.g., [123]) in the journal *Natural Product Reports*, highlighting the trend of minimal MNP research efforts on this phylum so far. Although only bryostatins are currently in clinical trials, potential future candidates that may reach clinical trials in the near future could belong to some of the less studied chemical classes, as reflected in this review (brominated alkaloids and neristatins, etc.). Most of the studied species reported here are cheilostomes that possess calcified skeletons (marine calcifiers). Surprisingly, the potential of skeletal organic matrix proteins in medical applications in calcifying marine invertebrates in general is still underexplored, in spite of it being considered a source of biochemical diversity [124]. Also, the usually small size and difficulty in identifying ctenostome bryozoans (uncalcified species) may have resulted in the group being overlooked in previous studies. Therefore, the isolation and characterization of their different classes of compounds as well as the focus on overlooked groups with a lack of natural mechanical defensive systems (e.g., ctenostomes) open up the possibility for future drug development.

Some limitations in bioactivity studies do exist and may be attributed to diverse reasons, such as the low amounts of compounds produced by the organisms (e.g., ~13 tons of *B. neritina* should be harvested to produce 18 g of the cancer candidate [125]), and the risk of overexploitation and habitat loss due to the collection of large number of specimens, especially when studying bryozoan species that can create reef structures (ecosystem engineers) in some regions and are particularly vulnerable to the combined effects of ocean acidification and global change [126]. Other difficulties in these kind of studies may also include the synthesis of specific compounds, the detection of low quantities of MNPs in microorganisms and hosts, the complex isolation and purification of skeletal proteins from marine calcifiers, the presence of toxins and inorganic salts in the extracts, and the variability of the chemical compounds (chemotype) produced by an organism under different environmental conditions. However, these limiting factors can be overcome by developing regulatory measures for bioprospecting to face the future expanding industry, especially in pristine areas with existing significant gaps in the current international legislation, like in Antarctica [127], by using chemical synthesis and advanced, sensitive, and selective analytical techniques (e.g., isolation, characterization, and separation of active compounds), by generating analogues with greater pharmacological activity (e.g., bryologs [128]), and by using high resolution mass spectrometry techniques (e.g., MALDI-TOF-imaging tools [129]), proteomic methods for identifying proteins in complex mixtures [124], and controlled aquaculture techniques (mariculture and sea-based farming). 

Among the bioactive compounds reported here, caulibugulones, eusynstyelamides, perfragilins, tambjamines, and bryostatins, as well as some sterols, are similar to those isolated from other marine invertebrate species, such as sponges, ascidians, and nudibranch mollusks. Therefore, the precise role of symbionts in the production of these bioactive compounds is also an exciting field for further research. To date, progress has been achieved on in vitro bacterial symbionts’ cultures (e.g., simulation of their natural environment and optimization of physical cultivation conditions [130]) for the supply of marine invertebrate-derived anticancer agents, although most bacterial symbionts associated with marine invertebrates still remain unculturable in the absence of their host (e.g., uncultured symbiotic bacterium *Endobugula sertula*). Furthermore, metagenomic sequencing is a powerful culture-independent tool that has already allowed for the identification of antitumor compounds produced by symbiotic microbes in some other marine invertebrates, such as an antitumor polyketide from a bacterial symbiont of the Japanese sponge *Theonella swinhoei* Gray, 1868 [131]. In this sponge, ten years later, Wilson and collaborators [132] also demonstrated that one uncultured bacterial symbiont produced almost all the polyketides and peptides that had been isolated from this sponge by genomic and biosynthetic studies. Therefore, cultivation-independent genetic strategies allow for the discovery of new anticancer drugs, including the approved anticancer drug Yondelis (ET-743, trabectedin). This tetrahydroisoquinoline alkaloid is produced by the uncultured symbiont *Candidatus* Endoecteinascidia frumentensis isolated from the Caribbean mangrove tunicate *E. turbinata* [133].

As bryozoans are almost ubiquitous, being present from the tropics to the poles and from the intertidal to the abyssal zone, larger chemical diversity (the result of diverse chemical interactions with other organisms and their environment) than other phyla is expected, making them a rich source of potential bioactive compounds. We have previously reported intraspecific variability in a range of biological activities (e.g., feeding repellence and antibacterial) for bryozoans depending on location and/or depth as an adaptive response to diverse abiotic and biotic factors and/or genetic or symbiotic variability [29,32]. In addition, these bryozoan species tested, together with the discovery of more than 20 new species [22,23], were collected in remote areas (polar regions and/or deep sea environments) using advanced diving and survey technologies. In fact, there are already preclinical studies of deep-sea-derived drugs (below 200 m) which are potentially useful in anticancer therapy [134]. Therefore, accurate taxonomic and geographic information of organisms producing bioactive compounds and the exploration of untapped geographical locations and overlooked taxa using advanced technologies are critical for further efficient bioprospecting of natural products from bryozoans and other understudied taxa. The close collaboration between researcher experts in multiple disciplines from academia (e.g., taxonomists, chemical ecologists, natural product chemists, molecular biologists, and microbiologists) and industry will help to overcome these difficulties in the field of drug discovery, together with the implementation of strategies for promoting the ecologically sustainable use and protection and conservation of marine biodiversity.

## Figures and Tables

**Table 1:** Compounds and cytotoxic activity against cancer cells described from bryozoans. 3T3: normal mice fibroblasts; 3T3-SV40: transformed mice fibroblasts; 768-O: renal carcinoma; A-2058: human melanoma cancer; BAEC: bovine aortic endothelial cell; B-16: mice melanoma; CaCo-2: human epithelial colorectal adenocarcinoma; CCRF-CEM: human leukemia; C6: rat histioblastome; HCT-116: human colon cancer cell line; Hela: human cervical cancer cell line; Hep-G2: hepatocellular carcinoma; HL-60: human plomyelocytic leukemia; HT-29: human colon carcinoma; IC-2^wt^: murine tumor; K1735-M2: murine melanoma; KB: human epidermoid carcinoma; KB/VJ-300: vincristine-resistant human cancer KB cell line; L-1210: murine leukemia; LNcaP: human prostate cancer; M-14: human melanoma; M-5076: reticulum cell sarcoma; MALME-3M: melanoma; MCF-7: human breast cancer; MDA-MB-435: human breast cancer; MDA-N: melanoma; MiaPaCa-2: human pancreatic cancer; MRC-5: human fibroblasts; NCI-60: a panel of 60 diverse human cancer cell lines of the U.S. National Cancer Institute; NCI-H23: human non-small cell lung cancer; P-388: murine lymphocytic leukemia; SGC-7901: human gastric carcinoma; SK-MEL-5: human melanoma; SK-N-SH: human neuroblastoma; U-937: histiocytic lymphoma.

*O. Cheilostomatida*	Geographical Area	Compounds	Activity against Cell Lines	References
Fam. Aspidostomatidae				
*Aspidostoma giganteum* (Busk, 1854)	Patagonia	***Alkaloids:*** *Aspidostomides* (Figure 1.2)	768-O	[45]
Fam. Bugulidae				
*Bugula neritina* (Linnaeus, 1758)	California, China, Gulf of Mexico	***Lactones:*** *Bryostatins* (Figure 2.10)	U-937, HL-60, P-388, B-16, K1735-M2, LNcaP, M-5076	[80,81,82,83,84,85,86,87,88,89,90,91,92,93,94,95,96,97,98,99,100,101,102,103,107]
		*Neristatins* (Figure 2.12)	P-388	[108]
*Caulibugula intermis* Harmer, 1926	Palau	***Alkaloids:*** *Caulibugulones* (Figure 1.3)	IC-2^wt^	[50]
*Virididentula (Bugula) dentata* (Lamouroux, 1816)	North Atlantic	***Alkaloids:*** *Tambjamines* (Figure 1.9)	CaCo-2	[76,77]
Fam. Catenicellidae				
*Paracribricellina (Cribricellina) cribraria* (Busk, 1852)	Australia, New Zealand	***Alkaloids:*** β-*Carboline alkaloids*	NCI-60, P-388	[45,46,47]
*Pterocella vesiculosa* (Lamarck, 1816)	New Zealand	***Alkaloids:*** β-*Carboline alkaloids*	P-388	[48]
		*Pterocellins* (Figure 1.6)	P-388, CCRF-CEM, MALME-3M, NCI-H23, M-14, SK-MEL-5, MDA-MB-435, MDA-N, Hela, and others	[68,69,70]
Fam. Cryptosulidae				
*Cryptosula pallasiana* (Moll, 1803)	China	***Lactones:*** *Other lactones*	HL-60, Hep-G2, SGC-7901	[111]
Fam. Flustridae				
*Flustra foliacea* (Linnaeus, 1758)	North Sea	***Alkaloids:*** *Deformylflustrabromine*	HCT-116	[67]
*Securiflustra securifrons* (Pallas, 1766)	Norway	***Alkaloids:*** *Securamines*	A-2058, HT-29, MCF-7, MRC-5	[66]
*Terminoflustra (Chartella) membranaceotruncata* (Smitt, 1868)	White Sea	***Alkaloids:*** *Terminoflustrindoles*	3T3, 3T3-SV40, SK-N-SH, C6, B-16, U-937	[78,79]
Fam. Membraniporidae				
*Biflustra perfragilis* MacGillivray, 1881	South Australia	***Alkaloids:*** *Perfragilins* (Figure 1.8)	P-388	[61]
Fam. Myriaporidae				
*Myriapora truncata* (Pallas, 1766)	Mediterranean	***Lactones:*** *Myriaporones* (Figure 2.11)	L-1210	[110]
Fam. Watersiporidae				
*Watersipora cucullata* (Busk, 1854)	Japan	***Ceramides***	DNA topoisomerase I enzyme	[112]
*Watersipora subtorquata* (d’Orbigny, 1852)	Japan	***Other compounds***	BAEC	[119]
**O. Ctenostomatida**				
Fam. Vesiculariidae				
*Amathia convoluta* (Lamarck, 1816)	Gulf of Mexico	***Alkaloids:*** *Convolutamides, convolutamydines and convolutamines* (Figure 1.4, Figure 1.5, Figure 1.7)	L-1210, KB, KB/VJ-300, P-388, HL-60, and other resistant lines	[51,52,53,54,55,56]
*Amathia wilsoni* Kirkpatrick, 1888	New Zealand	***Alkaloids:*** *Amathaspiramides* (Figure 1.1)	MiaPaCa-2	[41,42]

## Abbreviations

The following abbreviations are used in this manuscript:

3T3	normal mice fibroblasts
3T3-SV40	transformed mice fibroblasts
768-O	renal carcinoma
ADM	adriamycin
ALCL	anaplastic large cell lymphoma
A-2058	human melanoma cancer
BAEC	bovine aortic endothelial cell
bFGF	basic fibroblast growth factor
B-16	mice melanoma
CaCo-2	human epithelial colorectal adenocarcinoma
CCRF-CEM	human leukemia
C6	rat histioblastome
HCT-116	human colon cancer cell line
Hela	human cervical cancer cell line
Hep-G2	human hepatocellular carcinoma
HL-60	human plomyelocytic leukemia
HT-29	human colon carcinoma
IC-2^wt^	murine tumor
PKC	protein kinase C
K1735-M2	murine melanoma
KB	human epidermoid carcinoma
KB/VJ-300	vincristine-resistant human cancer KB cell line
L-1210	murine leukemia
LNcaP	human prostate cancer
M-14	human melanoma
M-5076	reticulum cell sarcoma
MALME-3M	melanoma
MB-231	breast carcinoma
MCF-7	human breast cancer
MDA-MB-435	human breast cancer
MDA-N	melanoma
MiaPaCa-2	human pancreatic cancer
MNP	marine natural products
MRC-5	human fibroblasts
MV4-11	acute myeloid leukemia
NCI-60	a panel of 60 diverse human cancer cell lines of the U.S. National Cancer Institute
NCI-H23	human non-small cell lung cancer
NCI-H460	human non-small cell lung cancer
P-388	murine lymphocytic leukemia
PC-3	human prostate cancer
SF-268	central nervous system cancer
SGC-7901	human gastric carcinoma
SK-MEL-5	human melanoma
SK-N-SH	human neuroblastome
U-937	histiocytic lymphoma
VCR	vincristine
